# Perfect Matchings with Crossings

**DOI:** 10.1007/s00453-023-01147-7

**Published:** 2023-07-18

**Authors:** Oswin Aichholzer, Ruy Fabila-Monroy, Philipp Kindermann, Irene Parada, Rosna Paul, Daniel Perz, Patrick Schnider, Birgit Vogtenhuber

**Affiliations:** 1 Institute of Software Technology, Graz University of Technology, Inffeldgasse 16b, 8010 Graz, Austria; 2 Departamento de Matemáticas, Cinvestav, Av Instituto Politécnico Nacional 2508, 07360 Ciudad de México, México; 3 Fachbereich IV – Informatikwissenschaften, Universität Trier, Universitätsring 15, 54296 Trier, Germany; 4 Departament de Matemàtiques, Universitat Politècnica de Catalunya, Edifici Omega, Jordi Girona 1–3, 08034 Barcelona, Catalunya Spain; 5 Department of Mathematical Sciences, University of Copenhagen, Universitetsparken 5, 2100 Copenhagen, Denmark

**Keywords:** Perfect matchings, Crossings, Geometric graphs, Combinatorial geometry, Order types

## Abstract

For sets of *n* points, *n* even, in general position in the plane, we consider straight-line drawings of perfect matchings on them. It is well known that such sets admit at least $C_{n/2}$ different plane perfect matchings, where $C_{n/2}$ is the *n*/2-th Catalan number. Generalizing this result we are interested in the number of drawings of perfect matchings which have *k* crossings. We show the following results. (1) For every $k\le \frac{1}{64}n^2-\frac{35}{32}n\sqrt{n}+\frac{1225}{64}n$, any set with *n* points, *n* sufficiently large, admits a perfect matching with exactly *k* crossings. (2) There exist sets of *n* points where every perfect matching has at most $\frac{5}{72}n^2-\frac{n}{4}$ crossings. (3) The number of perfect matchings with at most *k* crossings is superexponential in *n* if *k* is superlinear in *n*. (4) Point sets in convex position minimize the number of perfect matchings with at most *k* crossings for $k=0,1,2$, and maximize the number of perfect matchings with $$\left( {\begin{array}{c}n/2\\ 2\end{array}}\right) $$ crossings and with $${\left( {\begin{array}{c}n/2\\ 2\end{array}}\right) }\!-\!1$$ crossings.

## Introduction

The question of how many different plane (that is, crossing-free) straight-line perfect matchings can be drawn on a point set *P* in general position (that is, no three points are colinear) has been extensively studied; see for example [3–7]. It is known that $n=2m$ points in general position admit at least $C_m$ plane perfect matchings, where $$C_m=\frac{1}{m+1}{\left( {\begin{array}{c}2\,m\\ m\end{array}}\right) } \in 2^{\Theta (n)}$$ is the *m*-th *Catalan number*. This bound is tight, as point sets of size *n* in convex position (for short, *convex point sets*) allow exactly $C_{n/2}=C_m$ plane straight-line perfect matchings, and (almost) all other point sets allow strictly more [3, 5]. On the other hand, the number of plane straight-line perfect matchings of any set of *n* points is bounded from above by $O(10.05^n)$ [6]. Finally, there exist point sets which allow $\Omega (3.09^n)$ plane straight-line perfect matchings [4].

If we allow crossings, then we can draw every possible perfect matching. On *n* vertices there exist $(n-1)!!\in 2^{\Theta (n\log n)}$ such matchings [8], each having at most $$\left( {\begin{array}{c}n/2\\ 2\end{array}}\right) \in O(n^2)$$ crossings. Given *n*, the question of the existence of perfect matchings with exactly $$\mu =\left( {\begin{array}{c}n/2\\ 2\end{array}}\right) $$ crossings belongs to an active area of research around the so-called crossing family problem (see for example [9, 10]). A *crossing family* of a point set is a set of pairwise crossing straight-line edges spanned by the point set. Only recently, it was shown that any set of *n* points in general position contains a crossing family of almost linear size, more precisely, of size $n^{1-o(1)}$ [11]. This was the first substantial improvement after 25 years. The previously best known bound was $\sqrt{n/12}$, published in 1994 [9]. As a related result, Pach and Solymosi [12] gave a complete characterization of point sets admitting a *perfect crossing family*, that is, a perfect matching of pairwise crossing edges. If we restrict considerations to convex point sets, there are several results on the distribution of crossings over all perfect matchings; see for example [13–15]. However, not much is known about the existence or number of straight-line perfect matchings with *k* crossings for $0< k <\mu$. In this work, we make a substantial step towards filling this gap.

We analyze the number of straight-line perfect matchings with exactly or at most *k* crossings that a point set can admit. All considered point sets are in general position and have an even number of points. Additionally, we also assume that no two points lie on the same vertical or horizontal line. For brevity, we will from now on mostly omit the term straight-line. Further, *k-crossing* matchings and $(\le \!k)$*-crossing matchings* refer to perfect matchings with exactly *k* and at most *k* crossings, respectively.

We denote by ${\text {pm}} _k(P)$ the number of *k*-crossing matchings on a point set *P*, by ${\text {pm}}_{k}^{\max }(n)$ the maximum of ${\text {pm}} _k(P)$, taken over all sets *P* of *n* points, and by ${\text {pm}}_{k}^{\min }(n)$ the minimum of ${\text {pm}} _k(P)$, also taken over all sets *P* of *n* points. Similarly, we denote with ${\text {pm}} _{\le k}(P)$ the number of $(\le \!k)$-crossing matchings on a point set *P* and define ${\text {pm}}_{\le k}^{\max }(n)$ and ${\text {pm}}_{\le k}^{\min }(n)$ analogously as before. Finally, ${\text {pm}}_{k}^{{\text {conv}}}(n)$ is the number of *k*-crossing matchings on a set of *n* points in convex position.

**Contribution. ** We start by investigating matchings with exactly *k* crossings in Sect. 2. There we prove that for every $k\le \frac{1}{64}n^2-\frac{35}{32}n\sqrt{n}+\frac{1225}{64}n$, any set of *n* points with *n* sufficiently large admits a perfect matching with exactly *k* crossings (Theorem 1) and that there exist sets of *n* points where every perfect matching has at most $\frac{5}{72}n^2-\frac{n}{4}$ crossings (Theorem 2). We also investigate point sets where the possible numbers of crossings in perfect matchings are not consecutive. In Sect. 3 we then consider matchings with at most *k* crossings. We show that the number of perfect matchings with at most *k* crossings is superexponential in *n* if *k* is superlinear in *n* (Theorem 3), but only exponential if *k* is in $O(\frac{n}{\log n})$ (Corollary 1). Finally, in Sect. 4, we show that convex point sets are extremal in several aspects. More specifically, we show that convex point sets minimize the number of perfect matchings with at most *k* crossings for $k=0,1,2$ (Theorem 8), and maximize the number of perfect matchings with $\mu$ crossings and with $\mu \!-\!1$ crossings (Theorem 7).

## Perfect Matchings with Exactly *k* Crossings

In this section, we show that every set *P* of *n* points (with *n* sufficiently large) admits a *k*-crossing matching for every $0 \le k \le \frac{1}{64}n^2-\frac{35}{32}n\sqrt{n}+\frac{1225}{64}n$, while this is not the case for $k > \frac{5n^2}{72}-\frac{n}{4}$.

For even values of $n\le 10$, we have computed the numbers of perfect matchings with *k* crossings for all combinatorially different sets of *n* points using the order type data base; see [16] for details on order types. Table 1 lists the obtained numbers for sets of $n=6,8,$ and 10 points, from 0 up to the maximum number of $${\left( {\begin{array}{c}n/2\\ 2\end{array}}\right) }$$ crossings. We obtain the following observation.

**Table 1:** Numbers of *k*-crossing matchings for $n=6,8,10$ points

$n=6$				$n=8$				$n=10$			
*k*	min	conv	max	*k*	min	conv	max	*k*	min	conv	max
0	5	5	12	0	14	14	56	0	42	42	311
1	2	6	10	1	20	28	60	1	120	120	442
2	0	3	3	2	4	28	33	2	135	180	350
3	0	1	1	3	0	20	28	3	39	195	308
				4	0	10	10	4	0	165	165
				5	0	4	4	5	0	117	117
				6	0	1	1	6	0	70	72
								7	0	35	35
								8	0	15	15
								9	0	5	5
								10	0	1	1

For each number *k* of crossings, we display the minimum number of *k*-crossing matchings, the number of *k*-crossing matchings in the convex situation, and the maximum number of *k*-crossing matchings

### Observation 1

For any $k \le 3$, every set of at least 10 points admits a *k*-crossing matching.

For sufficiently large values of *n* we obtain the following result:

### Proposition 1

For any sufficiently large even value of *n*, every set *P* of *n* points admits a straight-line perfect matching with more than $\frac{77}{76}\frac{1}{64}n^2$ crossings.

### Proof

Consider the straight-line drawing of $K_n$ on *P*. Let *M* be a random perfect matching of $K_n$ chosen uniformly from the set of all perfect matchings of $K_n$. Let $\overline{M}$ be the corresponding straight-line drawing of *M* on *P*. For every set *T* of 4 points in *P*, let $X_T$ be the indicator random variable that has value equal to 1 if a pair of crossing edges of $\overline{M}$ has all four endpoints in *T* and 0 otherwise. Note that if *T* is not in convex position then $X_T=0$. Let *C* be the set of subsets of *P* of four points in convex position. If $T \in C$, then the expected value of $X_T$ is equal to the probability that *M* contains the only two crossing edges with endpoints in *T*. Hence, $E(X_T)=\frac{1}{(n-1)(n-3)}$. Let *X* be the number of pairs of edges of $\overline{M}$ that cross. We have that

$$\begin{aligned} E(X)=E\left( \sum _{T \in C} X_T \right) =\sum _{T \in C} E(X_T) =\frac{|C|}{(n-1)(n-3)}. \end{aligned}$$

Further, $|C |$ is also equal to the number of pairs of edges of $K_n$ that cross. The minimum number, $\overline{{\text {cr}}}(K_n)$, of pairs of edges that cross in any straight-line drawing of $K_n$ is called the *rectilinear crossing number* of $K_n$. It is known that $$\lim _{n \rightarrow \infty } \frac{\overline{\textrm{cr}}(K_n)}{\left( {\begin{array}{c}n\\ 4\end{array}}\right) }\,{=}\,q^*$$, for some positive constant $q^*$. Ábrego et al. [17] showed that $q^*>0.379972$. Thus, we have

1 $$\begin{aligned} E(X) \ge \frac{q^*\left( {\begin{array}{c}n\\ 4\end{array}}\right) }{(n-1)(n-3)} \ge \frac{0.379972 n (n-2)}{24} > \frac{77}{76} \frac{n^2}{64} \end{aligned}$$

for any sufficiently large value of *n*. The result follows. $\square$

We next show two technical lemmas which we afterwards use to prove in Theorem 1 that for sufficiently large *n* and any $0\le k\le \frac{1}{64} n^2-\frac{35}{32}n\sqrt{n}+\frac{1225}{64}n$, there always exists a perfect matching with *k* crossings.

### Lemma 1

Let *P* be a point set with *n* points and let *M* be a perfect matching on *P* with ${\text {cr}}(M)$ crossings. Let $0<k\le {\text {cr}}(M)$. Then *P* has a perfect matching $M'$ with $k-n+3 \le {\text {cr}}(M') \le k$ crossings.

### Proof

Let $M_0:=M$ and let $p_0,\ldots ,p_{n-1}$ be the points of *P* ordered from top to bottom. We obtain a matching $M_{i+1}$ from matching $M_{i}$ as follows. If $p_{2i}$ is matched to $p_{2i+1}$, then $M_{i+1} = M_i$. Otherwise, let $q_{2i},q_{2i+1}$ be the points of *P* matched to $p_{2i}$ and $p_{2i+1}$, respectively, in $M_{i}$. We replace the edges $(p_{2i},q_{2i})$ and $(p_{2i+1},q_{2i+1})$ by the edges $(p_{2i},p_{2i+1})$ and $(q_{2i},q_{2i+1})$. Note that the edges $(p_0,p_1),\ldots ,(p_{2i},p_{2i+1})$ have no crossing in $M_{i+1}$. Furthermore, the number of crossings of the edges $(p_{2i},q_{2i})$ and $(p_{2i+1},q_{2i+1})$ is at most $n-2i-3$ in $M_{i}$: in the worst case, each edge crosses all $((n-2i)/2)-1$ other edges, but the crossing between $(p_{2i+1},q_{2i+1})$ and $(p_{2i},q_{2i})$ is counted twice. Hence, we have ${\text {cr}}(M_{i+1}) \ge {\text {cr}}(M_{i})-n+2i+3 \ge {\text {cr}}(M_{i})-n+3$ and ${\text {cr}}(M_{n/2}) =0$. Then, the bound follows from choosing $M'=M_{i+1}$ such that $k \ge {\text {cr}}(M_{i+1}) \ge {\text {cr}}(M_i)-n+3 \ge k-n+3$. $\square$

### Lemma 2

For any sufficiently large even *n* and $0\le k\le \frac{9}{169} \frac{n^2}{64}$, it holds that ${\text {pm}}_{k}^{\min }(n) \ge 1$.

### Proof

Let $n_2:=10 \left\lfloor \frac{1}{13}n \right\rfloor$, and let  if  is even, or  otherwise. Note that $n_1+n_2\le n$, since *n* is even. We linearly separate the point set *P* into a point set $P_1$ consisting of the leftmost $n_1$ points and a point set $P_2$ consisting of the rightmost $n_2$ points.

Let $M_1$ be the matching of $P_1$ with the largest number of crossings. Then, by Proposition 1, $M_1$ has more than  crossings, where the last inequality holds for $n > 1330$.

By Lemma 1, $P_1$ has a matching $M_1'$ with $k-n_1+3 \le {\text {cr}}(M_1') \le k$ crossings, which we aim to extend to a *k*-crossing matching of *P*. To achieve this, we need to add exactly $\ell = k-{\text {cr}}(M_1')$ crossings during the completion. As $k-{\text {cr}}(M_1') \le n_1-3$, we have .

Next, we linearly separate $P_2$ into $\left\lfloor \frac{1}{13}n \right\rfloor$ sets of 10 points each. By Observation 1, every set of 10 points can be matched such that the matching has 0, 1, 2, or 3 crossings. Thus, we can find a matching of $P_2$ with exactly *x* crossings for every $0\le x\le ~3 \left\lfloor \frac{1}{13}n \right\rfloor$. Since $3\left\lfloor \frac{1}{13}n \right\rfloor \ge \frac{3}{13}n-3\ge \ell$, we can find a matching $M_2$ of $P_2$ with exactly $\ell$ crossings. By combining $M_2$ and $M_1'$, we get a matching $M=M_1'\cup M_2$ of *P* with exactly ${\text {cr}}(M) ={\text {cr}}(M_1') +{\text {cr}}(M_2) =k-\ell +\ell =k$ crossings. Finally, if $n_1+n_2<n$, we match the remaining points (which lie between $P_1$ and $P_2$) without additional crossings. $\square$

### Theorem 1

For any sufficiently large even *n* and $0\le k\le \frac{1}{64} n^2-\frac{35}{32}n\sqrt{n}+\frac{1225}{64}n$, it holds that ${\text {pm}}_{k}^{\min }(n) \ge 1$.

### Proof

We linearly separate the point set *P* into two parts; a point set $P_1$ consisting of the leftmost $n_1= n - 2\cdot \left\lfloor \frac{52}{3}\sqrt{n}\right\rfloor$ points and a point set $P_2$ consisting of the rightmost $n_2= 2\cdot \left\lfloor \frac{52}{3}\sqrt{n} \right\rfloor$ points. Note that $n_1$ is even since *n* is even.

Let $M_1$ be the matching of $P_1$ with the largest number of crossings; then $M_1$ has more than

$$\begin{aligned}{} & {} \frac{77}{76}\frac{1}{64}n_1^2> \frac{1}{64}\left( n - 2 \cdot \left\lfloor \frac{52}{3}\sqrt{n} \right\rfloor \right) ^2 {\mathop {\ge }\limits ^{\text {\tiny (n > 1225)}}} \frac{1}{64} \left( n-\frac{105}{3}\sqrt{n} \right) ^2 \\{} & {} \quad \ge \frac{1}{64}n^2-\frac{35}{32}n\sqrt{n}+\frac{1225}{64}n \end{aligned}$$

crossings by Proposition 1.

By Lemma 1, $P_1$ has a matching $M_1'$ with $k-(n - 2\cdot \left\lfloor \frac{52}{3}\sqrt{n}\right\rfloor )+3=k-n_1+3 \le {\text {cr}}(M_1') \le k$ crossings. Let $\ell = k-{\text {cr}}(M_1') \le n - 2\cdot \left\lfloor \frac{52}{3}\sqrt{n} \right\rfloor - 3$.

By Lemma 2, $P_2$ has a matching with exactly *x* crossings for every $0\le x \le \frac{9}{169} \frac{n_2^2}{64}$.

Note that

$$\begin{aligned} \frac{9}{169} \frac{n_2^2}{64}&= \frac{9}{169\cdot 64}{\left( 2\cdot \left\lfloor \frac{52}{3}\sqrt{n}\right\rfloor \right) ^2} \\&\ge \frac{9}{169\cdot 16}\left( \frac{52}{3}\sqrt{n} - 1 \right) ^2 \\&= n - \frac{9}{169\cdot 16}\left( 2\cdot \frac{52}{3}\sqrt{n} - 1 \right) \\&\ge n - \frac{9}{169\cdot 16} \left( 2\cdot \left\lfloor \frac{52}{3}\sqrt{n} \right\rfloor + 1\right) \\&\ge n- 2\cdot \left\lfloor \frac{52}{3}\sqrt{n}\right\rfloor - 3. \end{aligned}$$

Hence, there is a matching $M_2$ of $P_2$ with exactly $\ell$ crossings. This implies that there is a matching $M=M_1'\cup M_2$ of *P* with exactly ${\text {cr}}(M) ={\text {cr}}(M_1') +{\text {cr}}(M_2) =k-\ell +\ell =k$ crossings. $\square$

### Theorem 2

For any $n \equiv (0\!\! \mod 6)$ and $k>\frac{5n^2}{72} -\frac{n}{4}$, it holds that ${\text {pm}}_{k}^{\min }(n) = 0$.

**Fig. 1:**
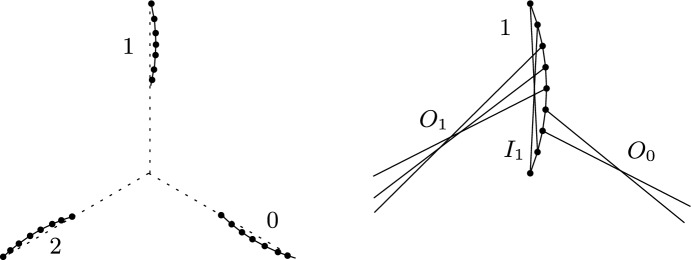
Illustration for the proof of Theorem 2: A point set *P* for which every perfect matching has $\le \frac{5n^2}{72}-\frac{n}{4}$ crossings (left). Interior ($I_1$) and outgoing ($O_0,O_1$) matching edges for *wing* 1 of *P* (right)

### Proof

For every $n \equiv (0\!\! \mod 6)$, we consider a specific set of *n* points, see Fig. 1. We call it a *windmill* consisting of three *wings*. Each wing is a set of *n*/3 points forming a flat convex chain that is arranged along a ray from the center of the windmill, where the rays for the three wings pairwise span angles of $2\pi /3$. The three wings are rotational symmetric copies of each other, and any line spanned by two points of one wing passes between the other two wings. We label the wings with indices 0,1, and 2 in counterclockwise order. In the following we consider all indices modulo 3. With $I_i$ we denote the collection of interior matching edges of wing *i*, that is, edges that match two points of this wing. With $O_i$ we denote the collection of outgoing matchings edges of wing *i*, that is, edges that match a point of wing *i* to wing $i+1$. Let ${{\iota }_{i}} = |I_i |$ and ${\text {o}}_{i} = |O_i |$ denote the cardinalities of these sets.

As we aim for an upper bound on the maximal possible number of crossings, we distribute the edges to maximize the crossings. We thus can assume that, for each wing *i*, the edges in $I_i$ and $O_i$ are arranged to maximize the possible crossings between them as well as within each of them (cf. the drawing on the right in Fig. 1). The number of crossings we can get this way is $$\sum _{i=0}^2 \left( {\left( {\begin{array}{c}{{\iota }_{i}} \\ 2\end{array}}\right) } + {\left( {\begin{array}{c}{\text {o}}_{i} \\ 2\end{array}}\right) }+{{\iota }_{i}} {\text {o}}_{i} \right) $$. So we have six parameters to maximize this sum. We get three equations for these parameters from the size of the wings, namely $2{{\iota }_{i}} + {\text {o}}_{i} + {\text {o}}_{i-1} =\frac{n}{3}$, $i \in \{1,2,3\}$. From this, we derive that ${\text {o}}_{i} =\frac{n}{6}+{{\iota }_{i-1}}- {{\iota }_{i}}- {{\iota }_{i+1}}$, and can thus express the number of crossings only using the numbers of interior matching edges, where $0 \le {{\iota }_{i}} \le \frac{n}{6}$. For the crossings formed by $I_i \cup O_i$, this gives

$$\begin{aligned}{} & {} {\left( {\begin{array}{c}{{\iota }_{i}} \\ 2\end{array}}\right) } + {\left( {\begin{array}{c}{\text {o}}_{i} \\ 2\end{array}}\right) }+ {{\iota }_{i}} {\text {o}}_{i} = {\left( {\begin{array}{c}{{\iota }_{i}} \\ 2\end{array}}\right) } + {\left( {\begin{array}{c}\frac{n}{6}+ {{\iota }_{i-1}}- {{\iota }_{i}}- {{\iota }_{i+1}} \\ 2\end{array}}\right) }+ {{\iota }_{i}} \left( \frac{n}{6}+ {{\iota }_{i-1}}- {{\iota }_{i}}- {{\iota }_{i+1}} \right) \\{} & {} \quad =\frac{n^2}{72}-\frac{n}{12}+\frac{n}{6}({{\iota }_{i-1}}- {{\iota }_{i+1}})+\frac{1}{2}\left( {{\iota }_{i+1}}- {{\iota }_{i-1}} + {{\iota }_{i-1}} ^2+ {{\iota }_{i+1}} ^2\right) -{{\iota }_{i-1}} {{\iota }_{i+1}}. \end{aligned}$$

Summing over all three wings gives $\frac{n^2}{24}-\frac{n}{4}+ {{\iota }_{0}} ^2+ {{\iota }_{1}} ^2+ {{\iota }_{2}} ^2- {{\iota }_{0}} {{\iota }_{1}}- {{\iota }_{0}} {{\iota }_{2}}- {{\iota }_{1}} {{\iota }_{2}}$ crossings.

By symmetry, we can assume w.l.o.g. that in the maximizing solution ${{\iota }_{0}} \ge {{\iota }_{1}}, {{\iota }_{2}}$ holds. Then, under the additional condition that all ${{\iota }_{i}}$ and ${\text {o}}_{i}$ have to be non-negative, the unique maximum is obtained for ${{\iota }_{0}} =\frac{n}{6}$ and ${{\iota }_{1}} = {{\iota }_{2}} =0$. This results in $\frac{n^2}{24}-\frac{n}{4}+\frac{n^2}{36}=\frac{5n^2}{72}-\frac{n}{4}$ crossings, which completes the proof. $\square$

### Gaps in the Number of Crossings

The above two theorems provide bounds on *k* such that for every point set and every $k'\le k$, the point set admits a $k'$-crossing matching. Now we are focusing on a fixed set *P* of *n* points and analyze for which values of *k* there exists a *k*-crossing matching on *P*. For some point sets *P*, there exist perfect matchings with all possible numbers *k* of crossings from 0 to $$\left( {\begin{array}{c}n/2\\ 2\end{array}}\right) $$. The following lemma states that this property holds for convex point sets.

#### Lemma 3

Every set of *n* points in convex position admits a *k*-crossing matching for every $$0 \le k \le \mu =\left( {\begin{array}{c}n/2\\ 2\end{array}}\right) $$.

#### Proof

**Fig. 2:**
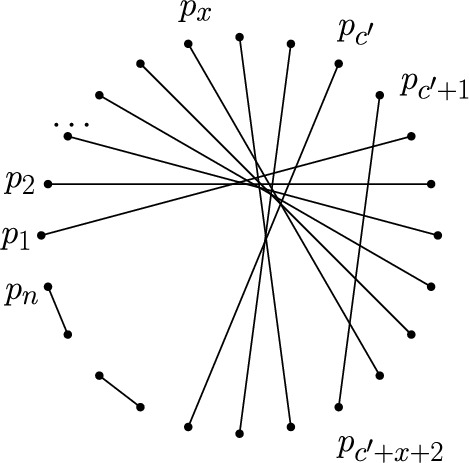
Proof of Lemma 3: Matching with 42 crossings obtained from a crossing family of size $c'=9$ plus $x=6$ extra crossings

Let *P* be a set of *n* points in convex position. Label the points in cyclic order from $p_1$ to $p_n$. A crossing family of size *c* is a set of *c* edges which pairwise cross and thus has $\frac{c(c-1)}{2}$ crossings. Let $c'$ be the largest integer such that $\frac{c'(c'-1)}{2} \le k$. To obtain *k* crossings in total observe that the number of crossings we still need to add to a crossing family of size $c'$ is $x=k-\frac{c'(c'-1)}{2} < c'$. We first construct the crossing family of size $c'$ by connecting the points $p_i$ and $p_{c'+i+1}$, for $i\in \{1,\ldots ,x\}$, and by connecting the points $p_i$ and $p_{c'+i+2}$, for $i\in \{x+1,\ldots ,c'\}$. See Fig. 2 for a depiction. Next we connect points $p_{c'+1}$ and $p_{c'+x+2}$ which were not yet connected within the crossing family and contribute the remaining *x* crossings. Finally, we match all remaining points (if any, with indices $2c'+3$ to *n*) without crossings. Then *M* has in total *k* crossings as desired. $\square$

**Fig. 3:**
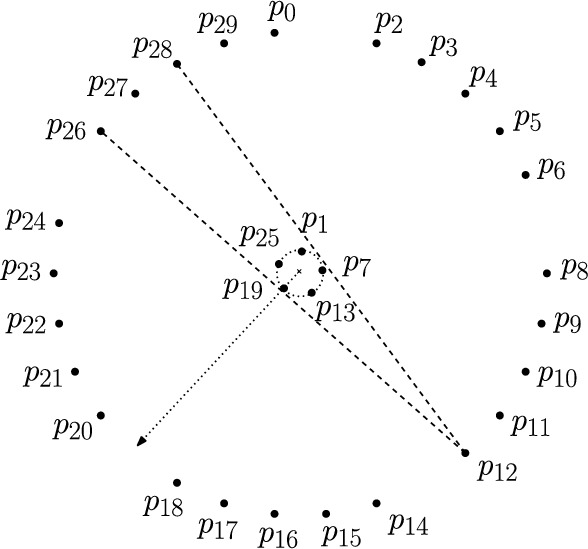
A set of 30 points with a perfect matching with $$\left( {\begin{array}{c}15\\ 2\end{array}}\right) =105$$ crossings, but with no perfect matching with $k\in \{103,104\}$ crossings. This result is obtained by direct computation

In contrast to Lemma 3, Fig. 3 gives an example of a set *P* of 30 points, that has a perfect matching with 105 crossings but does not have a perfect matching with 104 and 103 crossings. We say that *P* has a *gap* between 102 and 105, as it does not have a perfect matching with 103 and 104 crossings. The following proposition is inspired by the example in Fig 3. In its proof, we construct an infinite family of point sets with large gaps.

#### Proposition 2

For infinitely many values of *n*, there exists a set of *n* points which admits a matching with $$\mu =\left( {\begin{array}{c}n/2\\ 2\end{array}}\right) $$ crossings, but does not admit a matching with $\mu - i$ crossings for any $1\le i \le \frac{1}{4}(\sqrt{4n+1}-11)$.

#### Proof

We construct a point set $P_g$ with $n=4 g^2 + 6 g + 2$ points, where $g\ge 2$ is an integer, as follows. Consider a regular *n*-gon centered at the origin with points $p_0,\dots , p_{n-1}$. We consider all indices modulo *n*. Let $C_{\varepsilon }$ be a circle centered at origin and small enough such that no line spanned by $p_i$ and $p_{\frac{n}{2}+i \pm 1}$ for $1\le i \le \frac{n}{2}$ intersects $C_{\varepsilon }$. For $0 \le i \le 2g$, we push the points $p_{(2g+2)\cdot i}$ onto $C_{\varepsilon }$ along the ray from the origin through $p_{(2g+2)\cdot i}$; see Fig. 3 for a depiction with $g=2$. Note that the points $p_{(2\,g+2)\cdot i}$ with $0 \le i \le 2g$ form a regular $(2g+1)$-gon and are on $C_{\varepsilon }$. The set $P_g$ consists of those $2g+1$ points plus the remaining $n-2g-1$ vertices of the regular *n*-gon we started with. Obviously, $P_g$ spans a crossing family of size $\frac{n}{2}$, formed by the edges $(p_i, p_{i+\frac{n}{2}})$, $0\le i < \frac{n}{2}$, and hence admits a matching with $\mu$ crossings.

An edge connecting two points in $P_g$ is called *long* if it connects two points on the boundary of the convex hull of $P_g$. Otherwise it is called *short*.

#### Claim

If *M* has a long edge $(p_x,p_y)$ with $p_y \ne p_{x+\frac{n}{2}}$, then *M* has at most $\mu -g+1$ crossings.

*Proof of Claim.* We determine the maximal number of crossings of $(p_x,p_y)$. To do this, we count the number of points in the half planes $H_1$ and $H_2$ defined by the line spanned by $p_x$ and $p_y$. By construction, all points on $C_{\varepsilon }$ are in the half plane which contains more points of $P_g$, say $H_2$. Let $H'$ be the open half plane with $H_1 \subset H'$ that has the origin on its boundary (that is, the boundary of $H'$ is parallel to the line spanned by $p_x$ and $p_y$). Note that $H'$ contains at most $\frac{n}{2}$ points of $P_g$. Since the points on $C_{\varepsilon }$ form a regular $(2g+1)$-gon, every half plane with the origin on its boundary contains at least *g* points of $P_g$ on $C_{\varepsilon }$. Since $H_1 \subset H'$ and $H_1 \cap C_{\varepsilon } = \emptyset$, $H_1$ has at most $\frac{n}{2}-g$ points. Hence, $(p_x,p_y)$ has at most $\frac{n}{2}-g$ crossings.

The matching $M{\setminus } \{(p_x,p_y)\}$ has at most $\frac{(n/2-1)(n/2-2)}{2} = \frac{(n/2)^2-3(n/2)+2}{2}$ crossings. Hence, *M* has at most $\frac{(n/2)^2-3(n/2)+2}{2}+\frac{n}{2}-g=\mu -g+1$ crossings. $\blacksquare$

We prove by contradiction that there exists no matching with $\mu -i$ crossings for any $1\le i <g-1$. Assume that there exists a matching $M'$ with *k* crossings where $\mu -g+1< k < \mu$. The claim shows that all long edges of $M'$ are of the form $(p_x, p_x+\frac{n}{2})$ if $M'$ has more than $\mu -g+1$ crossings. Moreover, since there are $2g+1$ points on $C_{\varepsilon }$ and $4g^2+4g+1$ points on the boundary of the convex hull of $P_g$, there are at least $2g^2+g$ long edges of the form $(p_x, p_x+\frac{n}{2})$. Since there are only $2g^2+g$ long edges of the form $(p_x, p_x+\frac{n}{2})$, all those long edges are forced for any matching with more than $\mu -g+1$ crossings.

If there exists an edge of $M'$ between two points on $C_{\varepsilon }$, then there exists another long edge of the form $(p_x,p_y)$ with $p_y \ne p_{x+\frac{n}{2}}$, by the pigeonhole principle. It follows from the claim that any matching containing an edge between two points on $C_{\varepsilon }$ has at most $\mu -g+1$ crossings.

The remaining case is that every point on $C_{\varepsilon }$ is connected by a short matching edge to a point on the convex hull. Note that if $M'$ contains all short edges of the form $(p_x, p_{x+\frac{n}{2}})$, $p_x \in C_{\varepsilon }$, then $M'$ has exactly $\mu$ crossings. Hence $M'$ contains a short edge $(p_x, p_y)$ with $p_x \in C_{\varepsilon }$ and $y = x+\frac{n}{2}\pm r$ for some $1 \le r < \frac{n}{2}$. We consider $p_y = p_{x+\frac{n}{2}+ r}$ ($p_y = p_{x+\frac{n}{2}- r}$ follows analogously). Since $(p_x,p_{x+\frac{n}{2}})$ is a short edge and all long edges of the form $(p_x,p_{x+\frac{n}{2}})$ were forced in $M'$, $r>2g+1$. So the edge $(p_x, p_y)$ does not cross any of the line segments $(p_{x+i},p_{x+\frac{n}{2}+i})$ with $1\le i \le 2\,g+1$. On the other hand, 2*g* of the line segments $(p_{x+i},p_{x+\frac{n}{2}+i})$ correspond to the forced long edges. So $(p_x, p_y)$ has at most $\frac{n}{2}-2\,g$ crossings. Similar as above, it follows that $M'$ has at most $\mu -g+1$ crossings, a contradiction. $\square$

## Perfect Matchings with at Most *k* Crossings

We next show that if *k* is superlinear in *n*, then the number of $(\le \!k)$-crossing matchings is superexponential for every set of *n* points.

### Theorem 3

For any $k\in \omega (n)$, it holds that ${\text {pm}}_{\le k}^{\min }(n) \in 2^{\Omega (n \log (\frac{k}{n}))}$.

### Proof

Let *P* be a set of *n* points. By our assumption on point sets, no two points of *P* have the same *x*-coordinate. Process the points from left to right and partition them into $\lfloor \frac{n^2}{k} \rfloor$ groups $P_i$, with $i=1,2, \ldots , \lfloor \frac{n^2}{k} \rfloor$ of the same even number of points (plus one additional group $P'$ if *n* is not an even multiple of the number of groups). More exactly, if $\lfloor \frac{k}{n} \rfloor$ is even, then each group is of size $\lfloor \frac{k}{n} \rfloor$; otherwise it is of size$\lfloor \frac{k}{n} \rfloor -1$.

Consider first such a group $P_i$. As mentioned in the introduction, we can draw $2^{\Theta (\frac{k}{n}\log (\frac{k}{n}))}$ perfect matchings on $P_i$, and each of them has at most $${\left( {\begin{array}{c}\lfloor k/n \rfloor \\ 2\end{array}}\right) }< \lfloor \frac{k^2}{n^2} \rfloor $$ crossings. Now let $M'$ be an arbitrary but fixed plane matching of $P'$. Then the number of perfect matchings where all edges are either within some $P_i$ or in $M'$ is

2 $$\begin{aligned} \left( 2^{\Theta (\frac{k}{n}\log (\frac{k}{n}))}\right) ^{(\lfloor \frac{n^2}{k} \rfloor )}=2^{\Theta (n \log (\frac{k}{n}))}. \end{aligned}$$

Further, each such matching has at most $\lfloor \frac{n^2}{k} \rfloor \cdot \lfloor \frac{k^2}{n^2} \rfloor \le k$ crossings. Hence the number of $\le k$-crossing matchings of *P* is bounded from below by $2^{\Omega (n \log (\frac{k}{n}))}$. $\square$

Note that for a $(\le \!k)$-crossing matching, at most 4*k* points can be incident to crossing edges. Hence, the next theorem implies the upper bound on ${\text {pm}}_{\le k}^{\max }(n)$ as stated below in Corollary 1.

### Theorem 4

For any set *P* of *n* points, *n* even, and $0\le x\le n$, *x* even, let ${\text {pm}}^{x} (n)$ be the number of perfect matchings whose crossing edges are incident to at most *x* points. Then ${\text {pm}}^{x} (n)\in 2^{O(n + x\log x)}$.

### Proof

Consider some subset $P'\subset P$ of size *x*. We want to count the number of perfect matchings on *P* whose crossing edges are incident to points in $P'$. There are $2^{\Theta (x\log x)}$ perfect matchings on $P'$. We extend the matching on $P'$ to *P* by adding matching edges of $P{\setminus } P'$ such that the matching on $P{\setminus } P'$ is plane. As mentioned in the introduction, any point set in general position with *n* points, the number of plane perfect matchings is in $O(10.05^n) \in 2^{O(n)}$.

Thus there are at most $2^{O(n-x)}$ plane perfect matchings on $P{\setminus } P'$. Note, however, that edges of the matching on $P{\setminus } P'$ might intersect with edges from the matching on $P'$. But as we are only interested in an upper bound of the number of matchings where all edges with intersections are incident to points in $P'$, it is sufficient that these matchings are a subset of the matchings we consider in our construction. Finally, the choices for $P'$ are $$\left( {\begin{array}{c}n\\ x\end{array}}\right) \le 2^n$$. Hence, we get ${\text {pm}}^{x} (n)\in 2^n\cdot 2^{\Theta (x\log x)}\cdot 2^{O(n-x)} \subseteq 2^{O(n + x\log x)}.$ $\square$

### Corollary 1

For any even *n* and any *k*, it holds that ${\text {pm}}_{\le k}^{\max }(n)$ $\in 2^{O(n + k\log k)}$.

For $k\in \Omega (n)$, this bound is worse than the trivial upper bound from the number of all perfect matchings. For $k\in O(\frac{n}{\log n})$ we get a bound of $2^{O(n)}$, which is asymptotically tight.

##  Points in Convex Position

In this section, we study the number ${\text {pm}}_{k}^{{\text {conv}}}(n)$ of *k*-crossing matchings on a set of *n* points in convex position. Obviously, ${\text {pm}}_{k}^{\min }(n)$ $\le$ ${\text {pm}}_{k}^{{\text {conv}}}(n)$ $\le$ ${\text {pm}}_{k}^{\max }(n)$. It is well known that convex sets minimize the number of plane perfect matchings; see for example [5, 18]. Hence, we have ${\text {pm}}_{0}^{\min }(n)$ = ${\text {pm}}_{0}^{{\text {conv}}}(n)$. On the other hand, considering the maximum number $$\mu =\left( {\begin{array}{c}n/2\\ 2\end{array}}\right) $$ of crossings, we can show that for $k\in \{\mu ,\mu -1\}$, convex sets maximize the number of different *k*-crossing matchings. Precisely, the maximum number of $\mu$-crossing matchings and $(\mu -1)$-crossing matchings are 1 and *n*/2, respectively. Moreover, all sets of *n* points achieving these maximum numbers have exactly $\frac{n}{2}$ *halving edges* (edges that have $\frac{n-2}{2}$ points of the set on each side of their supporting line). We remark that, given *n* points, it is folklore that the minimum number of halving edges is *n*/2. The exact maximum number of halving edges is still unknown. The current best lower bound for the maximum number of halving edges on a point set of size *n* is $\Omega (ne^{\sqrt{\ln 4}\sqrt{\ln n}}/ \ln n)$ by Nivasch [19] and the current best upper bound is $O(n^{4/3})$ by Dey [20].

Next we state two results of Pach and Solymosi (adapted to our notation) that gives the existence and number of $\mu$-crossing matching w.r.t. the number of halving edges of the given point set.

### Theorem 5

(Theorem 1, [12]) A set of *n* points, *n* even, in general position in the plane admits a perfect crossing family if and only if it has precisely *n*/2 halving edges.

### Theorem 6

(Theorem 2, [12]) Any set of an even number of points in general position in the plane admits at most one perfect crossing family.

The following result is a consequence of Theorems 5 and 6.

### Corollary 2

For any even *n* and $$\mu =\left( {\begin{array}{c}n/2\\ 2\end{array}}\right) $$, it holds that ${\text {pm}}_{\mu }^{{\text {conv}}}(n) = {\text {pm}}_{\mu }^{\max }(n) =~1$.

### Proof

Let *P* be a set of *n* points in convex position labelled in cyclic order from $p_0$ to $p_{n-1}$. By Theorem 5, a point set *P* admits a $\mu$-crossing matching if and only if *P* has exactly $\frac{n}{2}$ halving edges. Thus, we construct our matching *M* to entirely consist of halving edges by connecting the points $p_i$ and $p_{\frac{n}{2}+i}$, for $i\in \{0,\ldots ,\frac{n}{2}-1\}$. Then any two edges of *M* cross and hence *M* has $\mu$ crossings. This is the only possible $\mu$-crossing matching, as Theorem 6 states that every set *P* of *n* points has at most one $\mu$-crossing matching. $\square$

### Theorem 7

For any even $n\ge 6$ and $$\mu -1 = \left( {\begin{array}{c}n/2\\ 2\end{array}}\right) -1$$, the following three statements hold.

1. $${\text {pm}}_{\mu -1}^{{\text {conv}}}(n) = {\text {pm}}_{\mu -1}^{\max }(n) = \frac{n}{2}.$$
2. Any set *P* of *n* points with exactly $\frac{n}{2}$ halving edges has ${\text {pm}} _{\mu -1}(P) \le \frac{n}{2}$.
3. Any set *P* of *n* points with more than $\frac{n}{2}$ halving edges has ${\text {pm}} _{\mu -1}(P) \le 2$.

### Proof

We will first show that any point set *P* with ${\text {pm}} _{\mu -1}(P) \ge 1$ either has exactly $\frac{n}{2}$ halving edges or has more than $\frac{n}{2}$ halving edges and ${\text {pm}} _{\mu -1}(P) \le 2$. Then, to complete the proof, we will show that any point set *P* with exactly $\frac{n}{2}$ halving edges has ${\text {pm}} _{\mu -1}(P) \le \frac{n}{2}$ and that $\frac{n}{2}$ is attained for convex point set.

Consider a set *P* of *n* points with ${\text {pm}} _{\mu -1}(P) \ge 1$ and a perfect matching *M* with $\mu -1$ crossings on *P*. Then *M* contains exactly one non-crossing pair of edges, say *e* and *f*. There are two ways on how the endpoints of *e* and *f* can be arranged: they can be in convex position (*e* and *f* are essentially “parallel”) or one endpoint is in the interior of the convex hull of the other three endpoints (one edge is “stabbing” the other). In this second case we denote the unique endpoint of *e* and *f* that is inside the convex hull as $p_c$.

**Case 1:** The endpoints of *e* and *f* are in convex position: In this case, we remove the edges *e* and *f* and add the diagonals of the convex quadrilateral defined by their endpoints to find a matching $M'$ with $\mu$ crossings. Since all other edges crossed *e* and *f*, they also cross the diagonals of this convex quadrilateral and thus the new edges. So we do not lose any crossing, but gain a crossing between *e* and *f*. Thus, by Theorem 5, the underlying point set has exactly *n*/2 different halving edges.

**Fig. 4:**
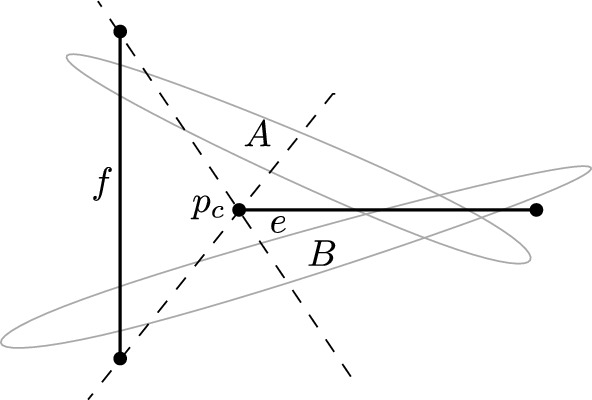
The case where the non-crossing edges *e* and *f* are in non-convex position

**Case 2: ** The point $p_c$ is in the interior of the convex hull of *e* and *f*: We assume w.l.o.g. that *e* is horizontal and that $p_c$ is the left endpoint of *e*. Moreover, *f* is vertical and to the left of $p_c$, and no other edges are horizontal or vertical; see Fig. 4 for an illustration. By the following claim, the underlying point set *P* has more than *n*/2 different halving edges.

### Claim

The point $p_c$ in the interior of the convex hull of *e* and *f* is incident to exactly 3 halving edges of *P* and all other points are incident to exactly one halving edge of *P*.

*Proof of Claim.* There are two types of edges other than *e* and *f* in the matching *M*: a family *A* of edges that cross *e* from above (edges with negative slope) and a family *B* of edges that cross *e* from below (edges with positive slope), where each of *A* and *B* might be empty. Clearly, all edges of *M* except *f* are halving edges of *P*. Now consider the lines through $p_c$ and the endpoints of *f*. As both *e* and *f* cross all the other edges of *M*, so does each of these lines. Hence, the two edges between $p_c$ and the endpoints of *f* are also halving edges of *P*. This shows that $p_c$ is incident to at least 3 halving edges, and every point of *P* is incident to at least one halving edge. Next we show that no other edge is a halving edge of *P*. Assume for a contradiction that some other edge *g* spanned by *P* is a halving edge as well. Let $M_e = M {{\setminus }} \{e\}$, $M_f = M {{\setminus }} \{f\}$, and $M_{e,f} = M {\setminus } \{e,f\}$. Similarly, let $P_e$, $P_f$, and $P_{e,f}$ be the point sets obtained from *P* by removing the endpoints of *e*, *f*, or *e* and *f*, respectively. Note that $M_e$, $M_f$, and $M_{e,f}$ are pairwise crossing perfect matchings of $P_e$, $P_f$, and $P_{e,f}$, respectively, and hence contain exactly all halving edges for their underlying point sets. Thus, *g* cannot cross *e* (or *f* or both), as otherwise *g* would be a halving edge of $P_e$ (or $P_f$ or $P_{e,f}$) and hence an edge of $M_e$ (or $M_f$ or $M_{e,f}$) contained in *M*. If *e* and *f* lie in the same closed halfspace of *g* then each edge of $M_{e,f}$ must have at least one endpoint in that halfspace as well, which gives a count of at least $2+\frac{n-4}{2} > \frac{n-2}{2}$ points of *P* in the open halfspace, a contradiction to *g* being a halving edge. If *e* and *f* lie in opposite closed halfspaces of *g*, then at most one endpoint of *g* is incident to *e* or *f* (as otherwise, *g* would be one of the three halving edges incident to $p_c$). Let $p_g$ be the endpoint of *g* that is not incident to *e* and *f*. The edge in *M* incident to $p_g$ lies in exactly one closed halfspace of *g* and hence crosses at most one of *e* and *f*, again a contradiction. $\blacksquare$

From this claim it follows that in Case 2 the point $p_c$ has to be the same for every matching of *P* with $\mu -1$ crossings. Further, in any such matching $p_c$ must be incident to a halving edge. Thus, for matchings other than *M*, it can only be matched with one of the endpoints of *f*. In particular, to obtain a matching with $\mu -1$ crossings, there are at most three choices for an edge incident to $p_c$, each choice uniquely determines the whole matching, and the edges in *A* and *B* must appear in any such matching.

As the edge incident to $p_c$ must cross the edges in both *A* and *B*, a different matching exists exactly if *A* or *B* are empty. If both *A* and *B* are empty, there are three possible matchings, but the underlying point set has only 4 points. If for $n\ge 6$ only one of *A* and *B* is empty and there are more than *n*/2 different halving edges, it follows that there can be at most two matchings with $\mu -1$ crossings.

It remains to show that if there are exactly *n*/2 different halving edges, then there are at most *n*/2 matchings with $\mu -1$ crossings. As argued above, only Case 1 can occur now, as in Case 2 there must be strictly more than *n*/2 different halving edges. A perfect crossing family (a perfect matching of pairwise crossing edges) defines a natural rotational order on its edges by sorting them by slopes. We call two edges neighbored if they are neighbored in this cyclic order. Similar to Case 1 above we can, for any matching with $\mu -1$ crossings, exchange the “parallel” edges with the diagonals of the corresponding convex quadrilateral. This gives a mapping from matchings with $\mu -1$ crossings to perfect crossing families where two neighboring edges are marked. Clearly, this mapping is an injection, showing that there are at most *n*/2 matchings with $\mu -1$ crossings. This mapping turns into a bijection when we have a convex point set and thus gives exactly *n*/2 such matchings. $\square$

### Remark 1

**Fig. 5:**
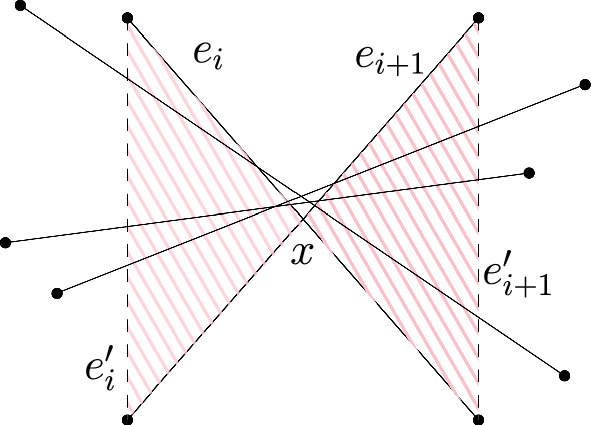
The pink shaded area represents the butterfly region of $e_i$ and $e_{i+1}$. If the butterfly region is empty of points, then any edge that crosses $e_i$ and $e_{i+1}$ also crosses $e_i'$ and $e_{i+1}'$

While Fig. 3 gives an example of a point set of size *n* with exactly *n*/2 halving edges that does not admit a perfect matching with $\mu -1$ crossings, we can characterize point sets that have exactly *n*/2 perfect matchings with $\mu -1$ crossings as follows. From the proof of Theorem 7 it is clear that such point sets have exactly *n*/2 halving edges and thus admit a perfect crossing family. Let $e_i$ and $e_{i+1}$ be two neighboring edges (w.r.t. the rotational ordering by the slope of the edges) of this perfect crossing family which cross at *x*. We construct the butterfly region of $e_i$ and $e_{i+1}$ by considering the convex quadrilateral *Q* formed by the endpoints of $e_i$ and $e_{i+1}$. Seen from *x* there are four wedges within *Q*. If for every edge of the perfect crossing family, except $e_i$ and $e_{i+1}$, we translate its supporting line such that it passes through *x*, then there are two wedges $W_1$ and $W_2$ where all these lines go trough – the other two wedges are empty since $e_i$ and $e_{i+1}$ are neighbored in the rotational order by slope. The union of the interior of $W_1$ and $W_2$ is the *butterfly region* of $e_i$ and $e_{i+1}$, see Fig 5. If this butterfly region is empty of any points of the given point set, then we can obtain a $(\mu -1)$-crossing matching by replacing $e_i, e_{i+1}$ with the two non-crossing edges that still cross all other edges ($e_i'$ and $e_{i+1}'$ in Fig. 5). Consequently, if the butterfly regions of all pairs of neighboring edges of a perfect crossing family are empty, then the underlying point set has exactly *n*/2 perfect matchings with $\mu -1$ crossings. So for $n \ge 8$ there are also non-convex point sets with *n*/2 perfect matchings with $\mu -1$ crossings.

**Fig. 6:**
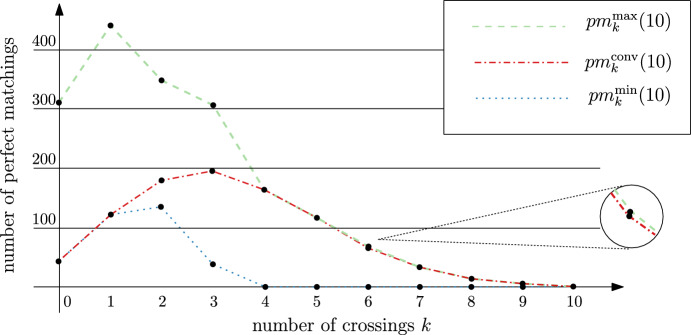
Comparison of ${\text {pm}}_{k}^{\min }(n)$, ${\text {pm}}_{k}^{\max }(n)$, and ${\text {pm}}_{k}^{{\text {conv}}}(n)$ for $n=10$ and $0 \le k \le 10$

It is natural to ask for which values of *k* and *n* it holds that ${\text {pm}}_{k}^{{\text {conv}}}(n) \in \{{\text {pm}}_{k}^{\min }(n), {\text {pm}}_{k}^{\max }(n) \}$; exhaustive computations for all point sets of small size indicate that this might be true for more than just $k\in \{0,\mu -1,\mu \}$. In Fig. 6, which is a graphical representation of Table 1 for $n=10$, we can see that ${\text {pm}}_{k}^{{\text {conv}}}(n) = {\text {pm}}_{k}^{\max }(n)$ for $4 \le k \le 10$, except for $k=6$. By analyzing this special case we found that the *wheel set* of size 10, where 9 points form a regular 9-gon with the remaining point in its center, is the unique point set that has the maximum of 72 perfect matchings with 6 crossings (whereas the convex set has only 70 perfect matchings with 6 crossings). By further examining wheel sets of small size we conjecture that they maximize the number of perfect matchings with $\frac{(n-2)(n-4)}{8}$ crossings, and that this maximum is $(n-1) 2^{\frac{n}{2} -2}$. For $k> \frac{(n-2)(n-4)}{8}$ crossings it still might hold that ${\text {pm}}_{k}^{{\text {conv}}}(n) = {\text {pm}}_{k}^{\max }(n)$.

As a variant of the above question, we consider for which values of *n* and *k* the convex set minimizes the number of matchings with at most *k* crossings, that is, ${\text {pm}}_{\le k}^{{\text {conv}}}(n)$ = ${\text {pm}}_{\le k}^{\min }(n)$? In the following, we prove the statement for any *n* and $k \le 2$. We start by showing a useful technical lemma.

We remark that the next lemma follows from variants of the Ham Sandwich Theorem. For self-containment, we give a short argumentation here.

### Lemma 4

Let $S =S_1 \cup S_2$ be a point set in general position in which $S_1$ and $S_2$ are separated by a line *h*. W.l.o.g. let *h* be horizontal, $S_1$ above *h* and $S_2$ below *h*. Then for any $a,b,c,d \in \mathbb {N}_0$, $a+b=|S_1 |-1$ and $c+d=|S_2 |-1$, there is a unique pair of points $p_1 \in S_1$ and $p_2 \in S_2$ such that the supporting line $\ell$ spanned by $p_1$ and $p_2$ splits $S_1$ such that there are *a* points to the left of $\ell$ and *b* points to the right of $\ell$, and at the same time $\ell$ splits $S_2$ so that there are *c* points to the left of $\ell$ and *d* points to the right of $\ell$.

### Proof

Consider the points of $S_1$ ordered from left to right and let *p* be the point with index $a+1$ in this order. Let $\ell$ be the vertical line through *p* and note that $\ell$ splits $S_1$ in the required way. If $\ell$ has *c* points of $S_2$ to its left, we are done. W.l.o.g. assume that $\ell$ has more than *c* points of $S_2$ to its left, and start rotating $\ell$ clockwise around *p*. (The case that $\ell$ has less than *c* points of $S_2$ to its left can be handled analogously by rotating $\ell$ counterclockwise around *p*.) Whenever $\ell$ touches a point $q \in S_1$, we set $p=q$ and continue the rotation around the new point *p*. Note that the splitting of $S_1$ remains *a* : *b*. If $\ell$ touches a point $q \in S_2$, then the number of points of $S_2$ to the left of $\ell$ is reduced by 1. If this number is *c*, then we stop the process and set $p_1=p$ and $p_2=q$. Otherwise, we continue the rotation. Before $\ell$ becomes horizontal, there are no points of $S_2$ to the left of $\ell$, so the process terminates.

We now show that there is only one pair of points $p_1$ and $p_2$ that spans a line $\ell$ which splits the sets in the required way. Assume for the sake of contradiction that there are two such lines $\ell '$ and $\ell ''$. Then $\ell '$ and $\ell ''$ intersect at most once, w.l.o.g. above *h*. Further assume that the notation is such that below *h*, line $\ell '$ is to the left of $\ell ''$. If $\ell '$ has, as required, *c* points of $S_2$ to its left, then $\ell ''$ has at least $c+1$ points to its left, as also the point $q \in S_2$ which spans $\ell '$ is to the left of $\ell ''$; a contradiction. $\square$

### Theorem 8

For any even *n* and any $k \in \{0,1,2\}$, it holds that ${\text {pm}}_{\le k}^{\min }(n) = {\text {pm}}_{\le k}^{{\text {conv}}}(n).$

### Proof

Let $P_C$ be a set of *n* points in convex position and let *P* be a set of *n* points in general position. We prove the theorem by establishing an injective mapping from perfect matchings with at most *k* crossings on $P_C$ to those on *P*. Let *M* be a perfect matching with at most *k* crossings on $P_C$. From *M* we will construct a perfect matching $M'$ on *P* with at most *k* crossings. Let *v* be the leftmost point on $P_C$ and let *w* be the point of $P_C$ to which *v* is matched in *M*. The line $\ell =vw$ splits $P_C$ in an upper half *U* and a lower half *L*. Map *v* to the leftmost point $v'$ of *P*. Let $w' \in P$ be the unique point such that there are $|U |$ points of *P* above the supporting line $v'w'$ (call this subset $U'$) and $|L |$ points of *P* below it (call this subset $L'$). Add $v'w'$ as an edge to $M'$.

If the edge *vw* is not crossed in *M*, then we treat the smaller sets *U* and *L* of $P_C$ and their corresponding sets $U'$ and $L'$ of *P* recursively. Note that each of these subsets has an even number of points and that they are linearly separated in both *P* and $P_C$, and can thus be treated independently.

If the edge *vw* is crossed in *M*, then we consider the leftmost crossing on it. Note that there exists a unique leftmost crossing on *vw*, as it cannot be a vertical line by the assumption on the point set. Let the crossing edge be *ul* with $u \in U$ and $l \in L$. By Lemma 4, there is a unique pair of points $u' \in U'$ and $l' \in L'$ such that the supporting line of $u'l'$ intersects the supporting line of $v'w'$ in *P* and the four resulting quadrants in *P* have the same cardinality as the corresponding quadrants in $P_C$. Note, however, that the edges $v'w'$ and $u'l'$ might not intersect (which is why we can show the mapping only with an upper bound on the number of crossings, and not with an exact bound).

**Fig. 7:**
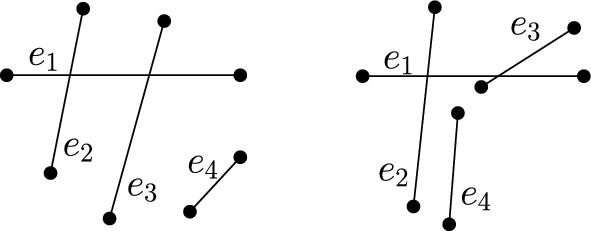
An illustration of the proof of Theorem 8 for $k=2$ crossings

The resulting four quadrants are disjoint convex regions. Hence, if none of the edges *vw* and *ul* is involved in the potential second crossing in *M*, then the subsets of points in these regions can be treated independently. If the edge *vw* (or the edge *ul*) contains a second crossing in *M*, then we repeat the just described procedure once more for the convex region formed by the two quadrants whose union contains the crossing. This results in a partition of the plane into six convex regions, each of which can be treated independently as $k \le 2$ (see Fig. 7).

Once all (at most two) crossings of the currently considered connected component of the matching *M* have been considered, we iterate on the remaining subsets as described above.

At the end of this construction, we obtain a perfect matching $M'$ in *P* which corresponds to the matching *M* in $P_C$ and has at most *k* crossings. As every step in this construction generated a unique matching edge, this gives the desired injective mapping. $\square$

**Fig. 8:**
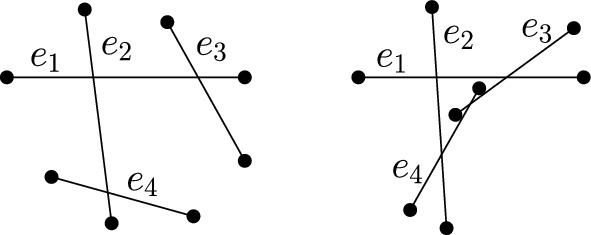
For $k=3$ crossings, the proof of Theorem 8 does not work: For the convex set (left) the given matching has three crossings. When drawing the edges on the second set (right) in the order given by the proof as indicated, an additional crossing occurs

Note that the above proof cannot be used to show the case for exactly *k* crossings. The reason is that if a matching of the convex set has a crossing, the according two edges might not cross in the matching of the general set. Moreover, the proof does also not work for $k>2$ crossings as it may contain cycles, where a cycle in a drawing is referred to as a closed region bounded by edge segments and crossings. Due to the presence of cycles, it might not be possible to treat the sub-quadrants independently. An example of such a situation is depicted in Fig. 8, where an additional crossing is generated. The crucial observation is that if only two crossings are allowed, such a situation cannot occur. (Actually, for the proof it is sufficient that any connected component of the drawing of the matching forms at most two crossings.)

## Conclusion

We have shown bounds for the number of perfect matchings with *k* crossings on point sets with an even number *n* of points. As with many other counting problems in discrete geometry and some decision problems on point sets in the plane, the computational complexity of deciding the existence of and counting the number of perfect matchings with *k* crossings is in general unknown.

We have shown that if $k\le \frac{1}{64}n^2-\frac{35}{32}n\sqrt{n}+\frac{1225}{64}n$, every set of *n* points, for *n* even and *n* sufficiently large, admits a perfect matching with *k* crossings. For those values of *k*, the existential question is therefore settled. On the other hand, as stated in [21], it is not even known whether counting the number of plane perfect matchings on a set of *n* points is hard, that is, #P-complete.

Given a set *P* of *n* points, the problem of deciding whether it admits a perfect matching with *k* crossings can be reformulated in terms of the intersection graph *G* of line segments connecting points in *P*. This graph contains a vertex for each segment connecting two points in *P* and edges connect intersecting segments, where an intersection is either a proper crossing or a common endpoint. We consider a 2-edge-coloring of this graph where edges corresponding to segments sharing an endpoint are colored blue and edges corresponding to crossing segments are colored red. The point set *P* admits a perfect matching with *k* crossings if and only if *G* has an induced subgraph with *n*/2 vertices, *k* red edges, and no blue edge. For general edge-colored segment intersection graphs, this problem is NP-complete by a reduction from the clique problem in such graphs [22]. However, the input parameters and the subclass of segment intersection graphs that we are interested in are very specific (though not so well understood), and it is therefore possible that the problem is polynomial-time solvable when restricted to these specific instances.

### Open Problems

In light of the above discussion, we point out the following open problems.

**Open Problem 1:** Determine the computational complexity of deciding whether a given point set admits a perfect matching with *k* crossings.

**Open Problem 2:** Investigate whether the number of perfect matchings with exactly one crossing is minimized by point sets in convex position.

**Open Problem 3:** Determine for which values of *k*, the number of perfect matchings with exactly *k* crossings is maximized by point sets in convex position.

In this paper we have shown that Open Problem 3 is true for $$k\in ~\{\left( {\begin{array}{c}n/2\\ 2\end{array}}\right) , \left( {\begin{array}{c}n/2\\ 2\end{array}}\right) -1\}$$. As we have mentioned before it could be also true for $k> \frac{(n-2)(n-4)}{8}$.

## Data Availability

Not applicable.
